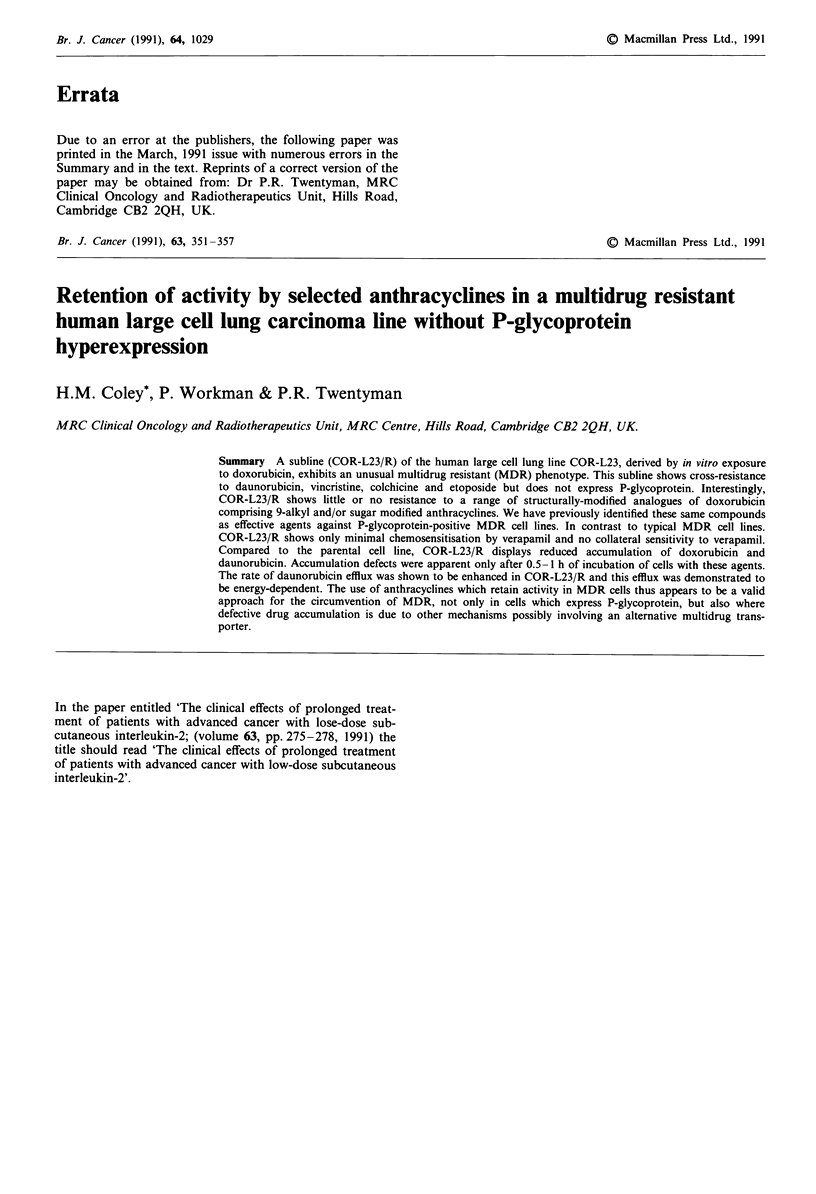# Errata

**Published:** 1991-06

**Authors:** 


 © Macmillan Press Ltd., 1991

### Errata

Due to an error at the publishers, the following paper was printed in the March, 1991 issue with numerous errors in the Summary and in the text. Reprints of a correct version of the paper may be obtained from: Dr P.R. Twentyman, MRC Clinical Oncology and Radiotherapeutics Unit, Hills Road, Cambridge CB2 2QH, UK.

*Br. J. Cancer* (1991), **63**, 351–357 © Macmillan Press Ltd., 1991

### Retention of activity by selected anthracyclines in a multidrug resistant human large cell lung carcinoma line without P-glycoprotein hyperexpression

H.M. Coley*, P. Workman & P.R. Twentyman

*MRC Clinical Oncology and Radiotherapeutics Unit, MRC Centre, Hills Road, Cambridge CB2 2QH, UK.*

**Summary** A subline (COR-L23/R) of the human large cell lung line COR-L23, derived by *in vitro* exposure to doxorubicin, exhibits an unusual multidrug resistant (MDR) phenotype. This subline shows cross-resistance to daunorubicin, vincristine, colchicine and etoposide but does not express P-glycoprotein. Interestingly, COR-L23/R shows little or no resistance to a range of structurally-modified analogues of doxorubicin comprising 9-alkyl and/or sugar modified anthracyclines. We have previously identified these same compounds as effective agents against P-glycoprotein-positive MDR cell lines. In contrast to typical MDR cell lines. COR-L23/R shows only minimal chemosensitisation by verapamil and no collateral sensitivity to verapamil. Compared to the parental cell line, COR-L23/R displays reduced accumulation of doxorubicin and daunorubicin. Accumulation defects were apparent only after 0.5–1 h of incubation of cells with these agents. The rate of daunorubicin efflux was shown to be enhanced in COR-L23/R and this efflux was demonstrated to be energy-dependent. The use of anthracyclines which retain activity in MDR cells thus appears to be a valid approach for the circumvention of MDR, not only in cells which express P-glycoprotein, but also where defective drug accumulation is due to other mechanisms possibly involving an alternative multidrug transporter.

In the paper entitled 'The clinical effects of prolonged treatment of patients with advanced cancer with lose-dose subcutaneous interleukin-2; (volume **63**, pp. 275–278, 1991) the title should read 'The clinical effects of prolonged treatment of patients with advanced cancer with low-dose subcutaneous interleukin-2'.